# The effectiveness of supervision strategies to improve health care provider practices in low- and middle-income countries: secondary analysis of a systematic review

**DOI:** 10.1186/s12960-021-00683-z

**Published:** 2022-01-06

**Authors:** Samantha Y. Rowe, Dennis Ross-Degnan, David H. Peters, Kathleen A. Holloway, Alexander K. Rowe

**Affiliations:** 1 CDC Foundation, Atlanta, USA; 2 Harvard Medical School, Boston, USA; 3 Harvard Pilgrim Health Care Institute, Boston, USA; 4 Department of International Health, Johns Hopkins Bloomberg School of Public Health, Baltimore, USA; 5 World Health Organization, Southeast Asia Regional Office, Delhi, India; 6 International Institute of Health Management Research, Jaipur, India; 7 Institute of Development Studies, University of Sussex, Brighton, UK; 8 Malaria Branch, Division of Parasitic Diseases and Malaria, Center for Global Health, Centers for Disease Control and Prevention, Atlanta, USA

**Keywords:** Developing countries, Health workers, Performance, Supervision, Quality improvement, Systematic review

## Abstract

**Background:**

Although supervision is a ubiquitous approach to support health programs and improve health care provider (HCP) performance in low- and middle-income countries (LMICs), quantitative evidence of its effects is unclear. The objectives of this study are to describe the effect of supervision strategies on HCP practices in LMICs and to identify attributes associated with greater effectiveness of routine supervision.

**Methods:**

We performed a secondary analysis of data on HCP practice outcomes (e.g., percentage of patients correctly treated) from a systematic review on improving HCP performance. The review included controlled trials and interrupted time series studies. We described distributions of effect sizes (defined as percentage-point [%-point] changes) for each supervision strategy. To identify attributes associated with supervision effectiveness, we performed random-effects linear regression modeling and examined studies that directly compared different approaches of routine supervision.

**Results:**

We analyzed data from 81 studies from 36 countries. For professional HCPs, such as nurses and physicians, primarily working at health facilities, routine supervision (median improvement when compared to controls: 10.7%-points; IQR: 9.9, 27.9) had similar effects on HCP practices as audit with feedback (median improvement: 10.1%-points; IQR: 6.2, 23.7). Two attributes were associated with greater mean effectiveness of routine supervision (*p* < 0.10): supervisors received supervision (by 8.8–11.5%-points), and supervisors participated in problem-solving with HCPs (by 14.2–20.8%-points). Training for supervisors and use of a checklist during supervision visits were not associated with effectiveness. The effects of supervision frequency (i.e., number of visits per year) and dose (i.e., the number of supervision visits during a study) were unclear. For lay HCPs, the effect of routine supervision was difficult to characterize because few studies existed, and effectiveness in those studies varied considerably. Evidence quality for all findings was low primarily because many studies had a high risk of bias.

**Conclusions:**

Although evidence is limited, to promote more effective supervision, our study supports supervising supervisors and having supervisors engage in problem-solving with HCPs. Supervision’s integral role in health systems in LMICs justifies a more deliberate research agenda to identify how to deliver supervision to optimize its effect on HCP practices.

**Supplementary Information:**

The online version contains supplementary material available at 10.1186/s12960-021-00683-z.

## Background

Health care providers (HCPs) are critical to increasing coverage of health interventions and producing better health outcomes. However, inadequate performance of HCPs in low-income and middle-income countries (LMICs) is common [1, 2]. Unsafe and ineffective medical care in LMICs has led to a considerable burden in terms of reduced productivity, disability-adjusted life years lost, and death [2–4].

For more than 40 years, supervision has been recommended as a strategy to support health programs and improve HCP performance in LMICs, where HCPs often work in isolated settings [5–11]. Kilminster et al. defined supervision as: “the provision of guidance and feedback on matters of personal, professional and educational development in the context of a trainee’s experience of providing safe and appropriate patient care” [12]. Bosch-Capblanch et al. emphasized that supervision helps connect peripheral health units and the district center [13]. Supervision typically includes a range of activities, usually at the supervisee’s workplace, such as problem-solving, reviewing records, and observing clinical practice [13, 14]. Many terms have been used to label supervision, such as routine supervision [13], managerial supervision [13], primary health care supervision, [14] enhanced supervision [15], supportive supervision [16, 17], and facilitative supervision [18].

Substantial resources have been used to support supervision. For example, the World Health Organization’s Programme for the Control of Diarrheal Diseases conducted supervisory skills training to health staff in more than 120 countries [19]. From 2017 to 2019, 69 of 72 country-specific annual plans of the President’s Malaria Initiative funded supervision [20–22]. For the 2018–2020 funding cycle, grant recipients of The Global Fund to Fight AIDS, Tuberculosis, and Malaria (Global Fund) budgeted US$311 million for supervision (personal communication; Rasheed Raji; Global Fund; June 1, 2021).

Although HCP supervision activities are ubiquitous in LMICs, evidence about their effectiveness is unclear. A recent analysis of data from an extensive systematic review of studies from LMICs (the Health Care Provider Performance Review, HCPPR) found that supervision had a moderate effect on HCP practices, widely varying across studies (median improvement: 14.8 percentage-points (%-points); range: –6.1, 56.3; interquartile range (IQR): 6.2, 25.2) [1]. That analysis, however, combined different strategies (e.g., routine supervision, audit with feedback, and peer review) with diverse implementation approaches (e.g., varying frequency and support for supervisors) into a single supervision category. Other reviews of supervision have key limitations, such as: providing only non-quantitative, narrative summaries; including few studies, few studies from LMICs, or studies with weak designs; or not accounting for the effect of non-supervisory co-interventions [13–16, 23, 24].

We performed a secondary analysis of HCPPR data to: (1) characterize the effectiveness of different supervision strategies, and (2) identify attributes associated with greater effectiveness of routine supervision. We present evidence that can help decision-makers select and design more effective supervision strategies, and we reveal important knowledge gaps about supervision effectiveness.

### Methods

This report uses the same methods as those used in an HCPPR-based analysis of training strategies [25]. We analyzed data from the HCPPR (PROSPERO registration CRD42016046154). Details of the HCPPR’s inclusion criteria, literature search, data abstraction, risk of bias assessment, effect size estimation, and assessment of publication bias have been published elsewhere [1, 26]. A summary is presented below.

### Inclusion criteria

The HCPPR included published and unpublished studies from LMICs in the public and private sectors that quantitatively evaluated a strategy to improve HCP performance. HCPs were broadly defined as hospital-, health facility-, or community-based health workers; pharmacists; and shopkeepers who sell medicines. Eligible study designs included pre- versus post-intervention studies with a randomized or non-randomized comparison group, post-intervention only studies with a randomized comparison group, and interrupted time series (ITS). The HCPPR included studies on any health condition in any language.

For this report, we only included studies that tested strategies with an HCP supervision-related component, although many strategies also had other intervention components. Additionally, we only analyzed HCP practice outcomes (e.g., patient assessment, diagnosis, treatment, counseling, and referral). These outcomes, which are typically the focus of supervision, were the most frequent ones studied in the HCPPR.

### Literature search and data abstraction

The HCPPR searched 52 electronic databases for published studies and 58 document inventories and websites for unpublished studies from the 1960s to 2016. The literature search also involved screening personal libraries and bibliographies from previous reviews and asking colleagues for unpublished studies.

To identify eligible reports, titles and abstracts were screened, and when necessary, a report’s full text was reviewed. Data were abstracted independently by two investigators or research assistants using a standardized form. Discrepancies were resolved through discussion. Data elements included HCP type, improvement strategies, outcomes, effect sizes, and risk of bias domains. Study investigators were queried about details not available in study reports.

### Strategy definitions

The HCPPR coded the presence of 207 strategy components for each study arm exposed to an improvement strategy and grouped them into categories. For HCP supervision strategies, six specific categories were created (e.g., routine supervision) (Box 1, top part). Eleven other categories were more general (e.g., training, group problem-solving).

Box 1. Strategy definitions

**Table:** 

**Supervision strategies for health care providers (HCPs) (all categories are mutually exclusive)** 1. *Routine supervision* Interactions between HCPs and a supervisor (who often does not work at the HCPs’ worksite) that could involve checking documentation and commodities, assessing HCP clinical practices, providing feedback to HCPs, and problem-solving. If a study reported that “supervision” was a strategy component, then it was classified as routine supervision, even if few or no details on the specific supervision activities were provided 2. *Audit with in-person feedback* A summary of an HCP’s clinical performance, based on data often collected from patient records over a specified period, is given to HCPs verbally, in person. In-person feedback may be given to HCPs in a group setting or to HCPs individually 3. *Audit with written feedback* A summary of an HCP’s clinical performance, based on data often collected from patient records over a specified period, is given to HCPs in a written or electronic format, not in person 4. *Peer review* HCPs are evaluated by their co-workers 5. *HCP received support from non-supervisory staff* For example, after training, facilitators were available to HCPs to address questions 6. *Benchmarking* The process of gathering information about HCPs or health facilities (usually the best-performing HCPs or facilities) to establish a “benchmark”, comparing the performance of HCPs and facilities against the benchmark, and using this information to set goals for improving performance **Other strategy components** **(all categories are mutually exclusive)**^**a**^ 1. *Community support.* E.g., community health education or social marketing of health services 2. *Patient support.* E.g., patient health education via printed materials or home visits 3. *Strengthening infrastructure.* E.g., provision of medicines or equipment 4. *HCP-directed financial incentives.* E.g., performance-based payments 5. *Health system financing and other incentives.* E.g., insurance or reducing a consultation fee 6. *Regulation and governance.* E.g., accreditation system 7. *Group problem-solving. *E.g., collaborative improvement or group problem-solving with or without formal teams 8. *Training. *E.g., group in-service training or educational outreach visits 9. *Other management techniques that do not include group problem-solving and supervision. *E.g., HCP self-assessment or HCP group process that is not group problem-solving 10. *Printed information or job aid for HCPs that is not an integral part of another component.* E.g., pamphlet for HCP.^b^ 11. *Information and communication technology (includes mHealth and eHealth) for HCPs.* E.g., computerized decision aid or text message reminders sent to HCPs’ phone	

^a^Detailed definitions in Appendix 1 (pages 39–44) of [1]

^b^Other strategy components (especially training) often include printed information for HCPs; and in these cases, the printed information was not considered a separate component

### Risk of bias assessment

Risk of bias was categorized at the study level as low, moderate, high, or very high, based on guidance from the Cochrane Effective Practice and Organisation of Care Group [27]. Randomized studies, ITS, and non-randomized studies were initially categorized as low, moderate, and high risk of bias, respectively. Risk of bias domains (e.g., dataset completeness, balance in baseline outcome measurements, etc.) were then assessed. A study’s risk-of-bias category was dropped by one level for every applicable domain that was “not done” and for every two applicable domains that were “unclear”.

### Estimating effect sizes

The primary outcome measure was the effect size, which was defined as an absolute %-point change in an HCP practice outcome and calculated such that positive values indicate improvement. For study outcomes that decreased to indicate improvement (e.g., percentage of patients receiving unnecessary treatments), we multiplied effect sizes by –1. For non-ITS studies with percentage outcomes (e.g., percentage of patients treated correctly), effect sizes were calculated using Eq. 1. Effect sizes were based on the baseline value closest in time to the beginning of the strategy and the follow-up value furthest in time from the beginning of the strategy:

1 $${\text{Effect size = }}\left( {{\text{follow-up}} - {\text{baseline}}} \right)_{{{\text{intervention}}}} - \left( {{\text{follow-up}} - {\text{baseline}}} \right)_{{{\text{control}}}}$$

In non-ITS studies, for unbounded continuous outcomes (e.g., average consultation time per patient in minutes), effect sizes were calculated with Eq. 2. If the baseline value for either the intervention or control group equaled zero, the effect size was undefined and thus excluded:

2 $${\text{Effect size }} =100\% \times\left[ {\left( {\frac{{{\text{follow-up}} - {\text{baseline}}}}{{{\text{baseline}}}}} \right)_{{{\text{intervention}}}} - \left( {\frac{{{\text{follow-up}} - {\text{baseline}}}}{{{\text{baseline}}}}} \right)_{{{\text{control}}}} } \right]$$

For ITS studies, segmented linear regression modeling was performed to estimate a summary effect size that incorporated both level and trend effects [28]. The summary effect size was the outcome level at the mid-point of the follow-up period as predicted by the regression model minus a counterfactual value that equaled the outcome level based on the pre-intervention trend extended to the mid-point of the follow-up period.

### Analysis

For objective 1 (characterize effectiveness of supervision strategies), we analyzed five types of study comparisons (Box 2). To estimate strategy effectiveness, the effect size for each study comparison was defined as the median of all effect sizes (MES) within the comparison. For example, if a study had three outcomes (e.g., percentages of patients correctly assessed, diagnosed, and treated) and one effect size per outcome, the MES was the median of the three effect sizes. For each supervision strategy, the MES distribution was described with a median, IQR, minimum, and maximum. Results were stratified by outcome scale (percentage versus continuous), HCP cadre (professional [generally health facility-based health workers] versus lay [generally community health workers]), whether the supervision was combined with other intervention components, and study type (equivalency versus non-equivalency).

Box 2. Study comparisons used to characterize the effectiveness of different supervision strategies (objective 1)

**Table:** 

*Non-equivalency studies (success is an effect size with a large positive magnitude)* • Comparison of a supervision strategy^a^ alone^b^ versus a (no-intervention) control group • Comparison of one supervision strategy^a^ alone^b^ versus a different supervision strategy^a^ alone^b^ (e.g., “audit with in-person feedback” versus “audit with written feedback”) • Comparison of a supervision strategy^a^ combined with a specific group of other strategy components^c^ versus that same specific group of other strategy components^c^ (e.g., “routine supervision plus training” versus “training”) • Comparison of one supervision strategy^a^ combined with a specific group of other strategy components^c^ versus a different supervision strategy^a^ combined with that same specific group of other strategy components^c^ (e.g., “routine supervision plus training” versus “peer review plus training”) *Equivalency studies (success is an effect size close to zero)* • Comparison in a supervision-related study of a strategy versus a “gold standard” comparison group^d^	

^a^Any of the six supervision strategies listed in the top part of Box 1

^b^That is, not combined with other strategy components listed in the bottom part of Box 1

^c^One or more of the 11 other strategy components in the bottom part of Box 1

^d^Only one equivalency study was included in the analysis. In that study, at baseline, lay HCPs in two study arms received routine supervision plus reminders about making home visits; during the intervention period, the gold standard control arm continued receiving both supervision and reminders, and the intervention arm received only the reminders

For objective 2 (identify attributes associated with the effectiveness of routine supervision), we used two approaches. First, we examined head-to-head studies that directly compared different supervision approaches (e.g., monthly versus bimonthly supervision). Second, we used random-effects linear regression modeling on studies of supervision with different approaches versus a control group. The dependent variable was the effect size, and the independent variables are presented in Box 3. For a list of independent variables that we initially considered but later excluded because their data were highly unbalanced (i.e., one level of the variable had < 5 comparisons), see Additional file 1: Section 1, items 2 and 6. The modeling accounted for the clustering of multiple outcomes from the same study. The regression analysis was performed on three hierarchical databases: supervision alone (*N* = 9 studies), supervision with or without training (hereafter referred to as the “supervision/training” database) (*N* = 21 studies), and supervision with or without other intervention components (hereafter referred to as the “supervision/other” database) (*N* = 58 studies). We restricted this analysis to studies of professional HCPs, supervision frequency ≤ 12 visits per year (studies with missing frequency were included; > 12 visits per year were considered unfeasible for most programs), and percentage outcomes. When interpreting regression model results, we focused on regression coefficients with a *p*-value less than 0.10. Given that, depending on the analysis, the evidence base for routine supervision comprised only a small-to-moderate number of studies and the regression analyses were exploratory in nature, we agreed with Friedman et al. [29] that decision-makers might consider findings with *p*-values larger than the “traditional” cut-off level of 0.05 as precise enough to inform policies—an approach also supported from a statistical perspective of evidence strength [30].

Box 3. Variables in the models used to identify attributes associated with the effectiveness of routine supervision (objective 2)

**Table:** 

*Supervision attributes* • Supervisors participated in a group process with health care providers that involved discussing a problem and collaborating to find a solution • Supervisors received supervision • Supervisors received training • An explicit element of supervisory visits was that supervisors gave feedback to health care providers • Supervisors used a standard checklist during supervisory visits • Supervision frequency (i.e., number of visits per year). For studies with a duration that was not a multiple of 12 months, frequency was estimated as: number of visits during the study intervention period divided by the intervention period (in months) times 12 • Number of supervision visits during the study’s intervention period (i.e., supervision “dose”) *Confounders* • Baseline performance level • Time since supervision was conducted (in months)

To characterize cost, we analyzed strategies involving routine supervision, as these were tested by the largest number of studies. As studies varied in terms of numbers of HCPs supervised and supervision frequency (with more visits being more expensive), we calculated the cost per HCP per supervision visit during the study intervention period.

All analyses were performed with SAS, version 9.4 (SAS Institute, Inc., Cary, North Carolina). More methodological details are available in Additional file 1: Section 1.

## Results

### Literature search

The HCPPR screened 216 483 citations and included 2272 reports (Additional file 1: Figure S1). Of those, 165 reports were eligible for this analysis. These reports presented 338 effect sizes from 90 comparisons in 81 studies (see Additional file 1: Tables S1–S4 for sample size details and Additional file 2 for study-level details and citations). These studies were conducted in 36 LMICs and represented a diversity of methods, geographical settings, HCP types, work environments, health conditions, and practices (Additional file 1: Tables S7–S10). Only one of the 81 studies involved an equivalency comparison that included a gold standard control group (Box 2, footnote d; Additional file 1: Table S1, Note). Nearly two-thirds of studies (63.0%) had randomized designs, and 42.0% had a low or moderate risk of bias. The median follow-up time per study was 6.0 months (from 74 studies that reported follow-up time; IQR: 3.0–11.5), median number of health facilities per study was 23 (from 64 studies reporting health facility sample size; IQR: 11–75), and median number of HCPs per study was 92 (from 45 studies reporting HCP sample size; IQR: 43–168). Most studies (81.5%) were published since 2000. We found no evidence of publication bias (Additional file 1: Figure S2).

### Effectiveness of supervision strategies (objective 1)

Table 1 presents the effects of supervision strategies on the practices of professional HCPs. As most results are based on one or two study comparisons and thus have limited generalizability, our discussion focuses on strategies tested by at least three study comparisons (i.e., at least three comparisons with percentage outcomes or at least three comparisons with continuous outcomes). The following findings are supported by low-quality evidence primarily because many studies had a high risk of bias.

**Table 1:** Effectiveness of supervision strategies on the practices of professional health care providers

Strategies tested^a^		No. of study comparisons (risk of bias: low, moderate, high, very high)	Outcome scale	Median MES^b^, in %-points (IQR; range)
Intervention arm	Reference arm			
*Routine supervision*				
Routine supervision	Controls	9 (3, 1, 4, 1)	Percentage	10.7 (6.9, 27.9; 2.1, 67.8)
Routine supervision	Controls	2 (0, 1, 1, 0)	Continuous	–29.5 (NA; –90.4, 31.4)
Routine supervision plus other strategy components	Other strategy components	4 (0, 0, 2, 2)	Percentage	4.1 (NA; 0, 7.1)
Routine supervision plus other strategy components	Other strategy components	1 (0, 0, 1, 0)	Continuous	24.9 (NA; NA)
*Routine supervision combined with benchmarking*				
Routine supervision plus benchmarking plus other strategy components^c^	Other strategy components	1 (0, 0, 1, 0)	Percentage	2.2 (NA; NA)^d^
			Continuous	–0.6 (NA; NA)^d^
*Audit with in-person feedback*^e^				
Audit with in-person feedback	Controls	4 (1, 1, 2, 0)	Percentage	15.0 (NA; 2.4, 33.5)
Audit with in-person feedback	Controls	1 (0, 0, 0, 1)	Continuous	–3.0 (NA; NA)
Audit with in-person feedback plus other strategy components	Other strategy components	1 (1, 0, 0, 0)	Percentage	5.0 (NA; NA)
*Audit with in-person feedback combined with peer review*^e^				
Audit with in-person feedback plus peer review	Controls	1 (0, 0, 1, 0)	Percentage	19.0 (NA; NA)
*Audit with written feedback*^e^				
Audit with written feedback	Controls	2 (2, 0, 0, 0)	Continuous	17.4 (NA; 17.3, 17.5)
*Audit with written feedback combined with benchmarking*				
Audit with written feedback plus benchmarking plus other strategy components	Other strategy components	1 (0, 1, 0, 0)	Percentage	0.2 (NA; NA)^d^
			Continuous	19.1 (NA; NA)^d^
*Audit with in-person feedback combined with audit with written feedback*^e^				
Audit with in-person feedback plus audit with written feedback	Controls	2 (2, 0, 0, 0)	Percentage	10.1 (NA; 8.5, 11.7)
*Audit with in-person feedback versus audit with written feedback*				
Audit with in-person feedback	Audit with written feedback	1 (0, 0, 0, 1)	Percentage	22.2 (NA; NA)^d^
			Continuous	16.7 (NA; NA)^d^
*Peer review and support from non-supervisory staff*				
Peer review plus other strategy components	Other strategy components	1 (0, 1, 0, 0)	Percentage	3.6 (NA; NA)^d^
			Continuous	33.0 (NA; NA)^d^
Health care provider received support from non-supervisory staff plus other strategy components	Other strategy components	2 (0, 2, 0, 0)	Percentage	–7.3 (NA; –16.9, 2.4)

%-points percentage-points, IQR interquartile range, MES median effect size, NA not applicable

^a^See Boxes 1 and 2 for descriptions of the strategies and the comparisons, respectively. This table only includes comparisons from non-equivalency studies

^b^Effect sizes calculated as the intervention arm improvement minus reference arm improvement

^c^Other strategy components include audit with in-person and written feedback

^d^Results for the percentage and continuous outcomes in this row are from the same study

^e^For six study comparisons for percentage outcomes involving audit with in-person feedback alone or combined with written feedback: median MES = 10.1%-points; IQR = 6.2, 23.7; range = 2.4, 33.5. For seven study comparisons for percentage outcomes involving audit with in-person feedback alone or combined with either peer review or audit with written feedback: median MES = 11.7%-points; IQR = 6.2, 23.7; range = 2.4, 33.5

**Fig. 1:**
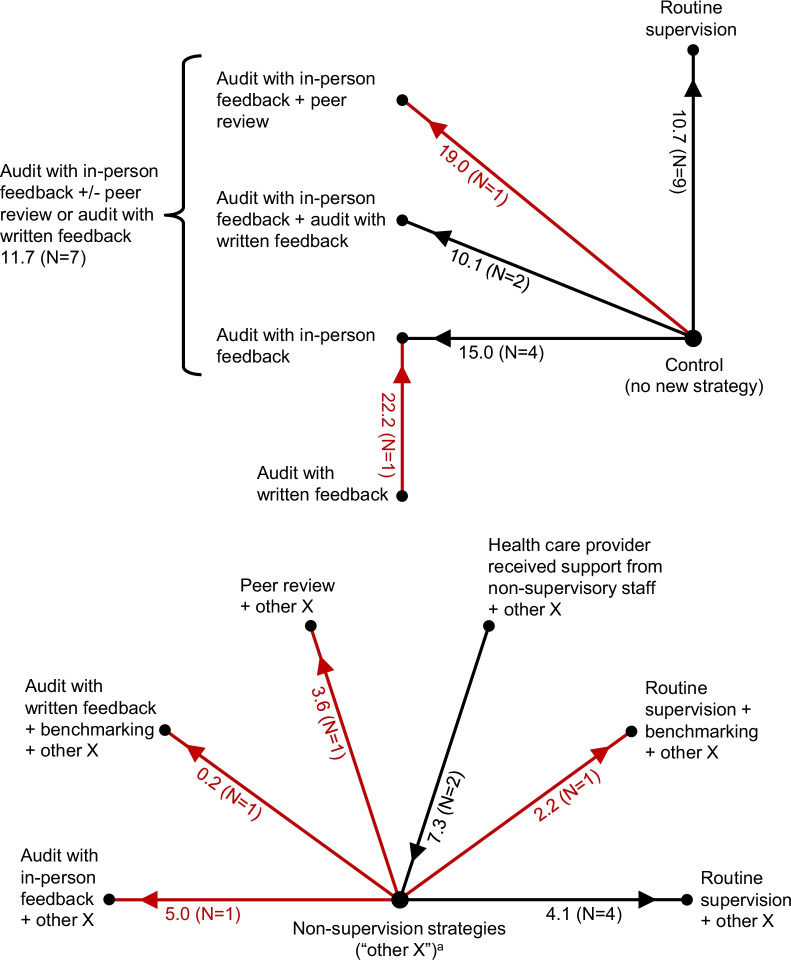
Effectiveness of supervision strategies for professional health care providers in low- and middle-income countries, as assessed with outcomes expressed as percentages. *N* = number of study comparisons. Red indicates results from a single study, which should be interpreted with caution. The numbers next to each spoke are the median of median effect sizes, in percentage-points, and (in parentheses) the number of study comparisons. For each comparison, the arrow points toward the study group with greater effectiveness. For example, routine supervision was more effective than controls by a median of 10.7 percentage-points. ^a^These are non-supervision strategy components (e.g., training) that could vary among study comparisons, but are the same for any two arms of a given study comparison (e.g., routine supervision plus training versus training)

For routine supervision alone, for percentage outcomes and when compared to controls, the median improvement in HCP practices was 10.7%-points (Table 1, row 1; Fig. 1). For example, for a percentage outcome with a typical baseline performance level of 40% and supervision effect of 10.7%-points, the post-supervision performance level would be 50.7%. Furthermore, supervision effects were very heterogeneous. One-quarter of MES values were relatively small (≤ 6.9%-points) and one-quarter were relatively large (27.9–67.8%-points). The marginal effect of routine supervision when added to other non-supervision strategy components was 4.1%-points (Table 1, row 3).

Audit-and-feedback alone, compared to controls, typically had an effect similar in magnitude to that of routine supervision. For percentage outcomes, the median effect of audit with in-person feedback alone was 15.0%-points (Table 1, row 6). For six study comparisons involving audit with in-person feedback alone or combined with written feedback (Table 1, rows 6 and 12), the median effect was 10.1%-points (IQR: 6.2, 23.7; Table 1, footnote e).

We found only four eligible studies (one with a moderate risk of bias, 3 with a high risk of bias) of supervision strategies to improve lay HCP practices (Additional file 1: Table S11). Most findings were supported by low-quality evidence. The effect of routine supervision was difficult to characterize, as results varied widely from the few studies that tested this strategy.

### Attributes associated with effectiveness of routine supervision (objective 2)

All results on supervision attributes are supported by low-quality evidence because many studies had a high risk of bias. See Additional file 1: Tables S5–S6 for sample sizes and risk-of-bias categories for all three modeling databases. Modeling of the “supervision alone” database included 9 comparisons from 9 studies, which are the same 9 comparisons in Table 1, row 1. Modeling of the supervision alone database found no supervision attribute with a univariable *p*-value < 0.10; thus, results of this database are not discussed further. Adjusted *R*^2^ values of the models for the other two databases (supervision/training and supervision/other) ranged from 0.11 to 0.27, indicating that they explain only a small amount of the variation in effect sizes.

Modeling of the supervision/other database showed that the mean effect of supervision in which supervisors received supervision was 8.8 to 11.5%-points higher than when supervisors had not received supervision (*p*-values: 0.051 to 0.097). The effect of supervisors participating in problem-solving with HCPs was large (14.2 to 20.8%-points, *p*-values: 0.032 to 0.098).

The effects of supervision frequency (i.e., number of visits per year) and dose (i.e., the number of supervision visits during a study) were unclear. One head-to-head study of lay HCPs with a low risk of bias found that monthly supervision was somewhat more effective than supervision every two months, by 7.5%-points. [31] However, the modeling results from studies of routine supervision among professional HCPs compared to no-intervention controls did not show a relationship between supervision dose and improvement in HCP practices  (from univariable models from the supervision only, supervision/training, and supervision/other databases: effects of –0.4 to –1.5%-points per additional supervision visit, *p* values: 0.12 to 0.50).

Training for supervisors, supervisor's use of a standard checklist, and explicit inclusion of feedback during supervision visits were not associated with effectiveness of routine supervision. Univariable modeling results for the effect of training for supervisors from the supervision only, supervision/training, and supervision/other databases were 14.3%-points (*p* = 0.17), 6.4 to 8.0%-points (*p*-values: 0.19 to 0.41), and –0.03 to 0.4%-points (*p*-values: 0.94 to 0.99), respectively. Univariable modeling results for the effect of a standard checklist from the three databases ranged from 4.1 to 5.8%-points (*p*-values: 0.27 to 0.54). Univariable modeling results for the effect of explicit inclusion of feedback from the supervision only and supervision/training databases ranged from 5.9 to 9.4%-points (*p*-values: 0.22 to 0.32), while multivariable models from the supervision/other database showed effects of 4.1 to 5.6%-points (*p*-values: 0.14 to 0.27; Additional File 1: Table S13).

Modeling of the supervision/training database showed that the mean effect of supervision increased by 0.68 to 0.91%-points per month (*p*-values: 0.012 to 0.081) since the beginning of supervision. However, modeling of the supervision/other database found that the effect of time was smaller (0.18%-points per month, *p* = 0.52). Increasing baseline HCP performance was consistently associated with decreasing supervision effectiveness, by 0.094 to 0.23%-points per 1%-point increase in baseline performance.

### Cost of routine supervision

Among 67 study arms from 62 studies of professional HCPs exposed to routine supervision, data on cost or from an economic evaluation of any type were available for only 25 arms, or 37.0%. Only 6 arms from 5 studies had data that allowed us to calculate cost per HCP per supervision visit. These 5 studies were from Africa, Asia, and Latin America. The median number of supervisory visits per arm was 2.5 visits (range: 1, 4). The median cost per HCP per supervisory visit was $46 (IQR: 25, 72; range: 10, 343). Cost was not related to study year, which ranged from 2001 to 2012.

Among 6 study arms from 5 studies among lay HCPs exposed to routine supervision, data on cost or from an economic evaluation of any type were available for 2 arms. Only 1 study arm had data that allowed us to calculate cost per HCP per supervision visit: $77 (from a 1992 study in Paraguay, a middle-income country).

## Discussion

In LMICs, health programs’ use of supervision to improve HCP performance is widespread and well-resourced, especially in recent years. We analyzed HCPPR data to compare the effectiveness of different supervision strategies and identify attributes associated with routine supervision effectiveness. Strengths of this study are that the data came from an extensive systematic review of evidence from LMICs, and we used multiple analytical approaches to gain a more comprehensive understanding of supervision effectiveness.

For professional HCPs, routine supervision was associated with moderate improvements in HCP practices when used as a sole intervention (median: 10.7%-points) and small marginal improvements when combined with other intervention components (median: 4.4%-points). Audit with feedback had similar effects. These findings were generally consistent with those from other reviews. The effect of supervision from Holloway et al., with all studies from LMICs, was 7.1%-points (personal communication from Kathleen Holloway, June 5, 2020) [32]. A review on audit with feedback (with 4 of 140 studies from LMICs) found a median effect of 4.3%-points for dichotomous outcomes [24]. It is likely no coincidence that the effects for supervision and audit with feedback are similar: although the labels for these strategies sound distinct, the intervention activities largely overlap.

The effect of benchmarking alone is unclear, as all studies with this strategy included other supervision-related intervention components. The effects of peer review and non-supervisory support for HCPs also are uncertain, as these strategies were tested in only 1 and 2 studies, respectively.

The effect of routine supervision for lay HCPs was difficult to characterize because few studies existed, and effectiveness in those studies varied considerably. A review by Gangwani et al. concluded that supervision “may enhance the quality of community health workers’ work” [17]; however, this review included some studies that were ineligible for our analysis because of weak study designs. Results from two trials from the Gangwani review that would have been eligible for our analysis (but were not included because they were published after the HCPPR literature search had ended), had they been added, would not have changed our conclusions (see Note on Additional file 1: Table S11).

We found two attributes associated with higher effects of routine supervision for professional HCPs: supervisors received supervision, and supervisors participated in problem-solving with HCPs. Providing supervision is difficult, with supervisors facing many challenges, such as inadequate management skills, non-supervision duties that leave insufficient time for supervision, and loss of effective supervisors due to staff turnover [13–15, 33]. These challenges remind us that supervisors are health workers too [33], and they need regular supportive guidance and feedback to help overcome barriers to effective implementation of supervision.

Involving HCPs in problem-solving, as in the “improvement collaborative” approach, has been associated with large improvements in HCP performance [1, 34–36], and joint problem-solving between a supervisor and supervisee is considered a helpful behavior [12, 14]. A review by Bailey et al. however, noted that problem-solving during supervision “did not necessarily translate into consistent improvements in clinical practice, unless the supervisor was considered as friendly and supportive” [16].

We found inconclusive results on the effects of supervision frequency and dose. Our analysis, however, was limited by: missing data on supervision frequency, potential reverse causality or confounding if supervisors made more visits to health facilities where improvements were more difficult to achieve, and potential dilution of effect if HCPs exposed to supervision were not the same HCPs surveyed [15]. Nevertheless, our results seem to reflect the current state of the literature. Two studies that performed within-study analyses found that increasing supervision dose was associated with better performance [37, 38], and a review of audit with feedback concluded that feedback might be more effective if it is provided more than once [24]. However, another review found that more intensive supervision (e.g., with more frequent visits) is not necessarily more beneficial [13].

Our results did not corroborate one review’s recommendation that training for supervisors would increase effectiveness [15]. Univariable modeling from several databases consistently found weak statistical evidence for the effect of training for supervisors.

Regarding the effect of supervision over time, we found improvements of 0.18 to 0.91%-points per month. Another analysis of HCPPR data by Arsenault et al. that examined the effect of time in a more nuanced fashion (using multiple follow-up time points per study) found inconsistent time trends for supervision: some analyses found positive time trends (mean improvements of 0.82 to 0.88%-points per month), while a key sensitivity analysis showed no improvement over time [34].

Our study’s finding about the association between baseline HCP performance and the effectiveness of routine supervision agreed with a review of audit with feedback, which concluded that feedback might be more effective when baseline performance is low [24].

The overall strength of evidence on supervision strategies to improve HCP practices is weak, and substantial knowledge gaps remain. Our understanding of supervision would benefit from additional studies using more rigorous designs and standardized methods to replicate key results (an essential part of the scientific method), investigate promising new supervision strategies, identify the optimal frequency of supervision, and expand the evidence base for lay HCPs (Box 4). Future studies should report details on supervision frequency, cost, context, and—of particular importance—the specific activities of the supervision process. Such process details could be used to classify and compare strategies more precisely in future reviews and thus facilitate decision-making by programs. Non-standardized strategy labeling is a challenge with quality improvement research in general, and researchers and implementors would be wise to move beyond the vague descriptors that are too often used for strategies such as supervision.

Box 4. Evidence-based recommendations for strengthening research on supervision strategies to improve health care provider practices in low-income and middle-income countries

**Table:** 

*Regarding topic areas, future research should focus on:* • Replicating studies of promising strategies tested with few studies (e.g., audit with in-person feedback plus peer review) • Head-to-head comparisons of key supervision strategies (e.g., routine supervision versus audit with feedback), strategy combinations (e.g., audit with feedback plus peer review versus audit with feedback alone) and supervision attributes (e.g., different supervision methods, such as involving supervisors in group problem-solving with HCPs, supervision of supervisors, and frequencies). Understanding the optimal frequency or dose of supervision in different contexts is an especially critical topic • Rigorous studies of supervision strategies to improve the practices of lay or community health workers • Better quantitative and qualitative understanding of how context influences strategy effectiveness *﻿Regarding methods, future research should:* • Use standardized methods, especially for outcomes, strategy description, implementation (including dose and fidelity), and characterization of study context • Prioritize head-to-head studies, which provide stronger evidence for comparing different supervision approaches • Have rigorous study designs, such as interrupted time series with a randomized comparison group, which reduce bias and show how effectiveness changes over time • Have follow-up periods that match the timeframe that programs require for improvements to be meaningful (e.g., at least 12 months) and include multiple measures of effect so changes (reductions or further improvements) in effectiveness over time can be quantified • Include assessments of strategy cost and cost-effectiveness • Be designed to better contribute to filling gaps in the evidence base about strategy choice and combinations of components^a^	

^a^Studies directly comparing two supervision approaches without other components are the easiest to interpret. However, given the generally moderate effect of supervision as a sole strategy, studies should include other enhancing components in both study arms (e.g., supervision approach A + training versus supervision approach B + training)

Key limitations of our analysis were that included studies had heterogeneous methods and contexts, high risk of bias, short follow-up periods, not all relevant supervision attributes were abstracted, missing values for supervision dose, potential misclassification of supervision attributes, and confounding from unmeasured factors. Modeling did not adjust for multiple comparisons, so the results reflect hypothesis screening rather than true hypothesis testing. Also, our interpretation of modeling results with a *p*-value less than 0.10 might have increased our chances of mistakenly concluding there was an effect, when truly there was not. Furthermore, our results had limited generalizability because most strategies were tested by few studies, and research settings often differ from real-world programs because they receive extra resources and technical assistance. This last point is especially important given the well-documented challenges of implementing routine supervision [33]. Details on these limitations are presented in Additional file 1: Box S1. The direction and magnitude of the biases that these limitations might introduce are difficult to determine.

Given these limitations, programs should not assume that the effect of a certain approach in our analyses will be the same in their specific context. As with any improvement strategy, we recommend that programs continually assess HCP performance to understand a supervision strategy’s effect [1].

## Conclusions

Although the evidence is limited, our study has characterized the effectiveness of several supervision strategies in LMICs and supports supervising supervisors and having supervisors engage in problem-solving with HCPs for more effective supervision. We also developed evidence-based recommendations for strengthening future research on supervision strategies. Supervision’s integral role in health systems in LMICs justifies a more deliberate research agenda to identify how to deliver supervision to optimize its effect on HCP practices, health programs, and health outcomes.

## Supplementary Information

**Additional file 1:** Methodological details and additional results.

**Additional file 2:** Characteristics of included studies.

## Data Availability

The datasets generated during and/or analyzed during the current study are available at the Health Care Provider Performance Review website, http://www.HCPperformancereview.org.

## Abbreviations

CI: Confidence interval
HCP: Health care provider
HCPPR: Health Care Provider Performance Review
IQR: Interquartile range
LMIC: Low- and middle-income country
