# Supplementary material for: The effectiveness of supervision strategies to improve health care provider practices in low- and middle-income countries: secondary analysis of a systematic review
Source: Hum Resour Health. 2022 Jan 6;20:1. doi: 10.1186/s12960-021-00683-z (PMC8734232; doi:10.1186/s12960-021-00683-z)
Supplement: Supplementary file 1 — Additional file 1: Methodological details and additional results. [file 12960_2021_683_MOESM1_ESM.docx]

**Additional File 1 for:**

**Rowe SY, Ross-Degnan D, Peters DH, Holloway KA, Rowe AK. The effectiveness of supervision strategies to improve health care provider practices in low- and middle-income countries: secondary analysis of a systematic review**

Section 1. Methodological details

Section 2. Additional results

Section 1. Methodological details

**Objective 1: Characterize the effectiveness of different supervision strategies**

- To define the non-supervision part of a strategy (i.e., the “other” strategy components), we used strategy definitions at the “component category” level, rather than the “individual component” level. Usually, the non-supervision parts of the strategy in two arms from the same study were identical at the individual component level (e.g., arm 1 = “provision of drug supply”, and arm 2 = “supervision + provision of drug supply”). On a few occasions, the individual strategy components between study arms did not exactly match; however, the categories of strategy components matched, and therefore, we classified them as the same. For example, in one study comparison: arm 1 had “poster for HCP + poster for community + drug supply” and arm 2 had “supervision + educational video for community + poster for HCP + poster for community + drug supply.” Even though the individual “community support” components were not exactly the same between the arms (Arm 1: poster for community vs. Arm 2: educational video + poster for community), both arms had components in the “community support” category so that the "other" non-supervision parts of the strategies were the same in both arms.
- Indirect analysis (true control comparisons) and direct analysis (head-to-head comparisons) results were presented together in a “network” diagram (Figure 1). However, formal network meta-analysis was not performed because it would not have added much to the simpler analysis used. Specifically (as can be seen in Figure 1, the main network (with the no-intervention reference group) is connected to only one additional strategy via a head-to-head comparison.

**Objective 2: Identify attributes of routine supervision associated with supervision effectiveness**

- Indirect analysis (modeling of effect sizes from study comparisons with a no-intervention control group)
  1. For each of the three databases of studies of routine supervision (i.e., supervision only, supervision +/- HCP training, supervision +/- other strategy components), we created a mixed model with a random-intercept (in which the cluster was the study) using a 3-step approach: 1) univariable analyses of individual supervision attributes; 2) attributes with univariable p-values < 0.10 were identified; and 3) step 2 attributes were included in a multivariable model. Unless otherwise noted, we used the SAS MIXED procedure with the "variance component" structure (i.e., the variance of each effect size within a study is different, and covariances between effect sizes is 0) and used the “residual maximum likelihood” method to estimate variance parameters.
  2. Some supervision attributes were excluded because they were highly unbalanced (i.e., one level of the attribute had <5 comparisons)
     1. Database of studies on supervision only. Seven indicator variables for supervision characteristics were excluded: whether the supervision was labeled as “supportive supervision” by the study investigators, whether the supervisor received financial incentive, whether the supervisor received non-financial incentives, whether the strategy involved a decrease in supervision, whether the supervision was implemented only once or it was ongoing, whether new guidelines for supervision were introduced, and whether supervisors participated in problem-solving with HCPs.
     2. Database of studies on supervision +/- HCP training (see Table D1). Seven indicator variables for supervision characteristics were excluded: whether the supervisor received financial incentive, whether the supervisor received non-financial incentives, whether the strategy involved a decrease in supervision, whether the supervision was implemented only once or it was ongoing, whether new guidelines for supervision were introduced, whether supervisors participated in problem-solving with HCPs, and whether supervisors were supervised.
     3. Database of studies on supervision +/- other strategy components (see Table D2). Four indicator variables for supervision characteristics were excluded: whether the supervisor received financial incentive, whether the supervisor received non-financial incentives, whether the strategy involved a decrease in supervision, and whether the supervision was implemented only once or it was ongoing.
  3. Attempts to add supervision attributes not included in the step 3 model resulted in unstable models. Out of concerns that more complex models might be over-specified, we only tested one set of additional models that included variables for baseline performance and time since supervision began, as they were known predictors of effect size. Thus, for each of the three databases, we tested two models (see Tables D1–D2).
     1. Model with no predictors forced into the model
     2. Model with baseline performance and time since supervision began forced into the model
  4. An additional modeling approach involved forcing the “supervisor provided feedback” variable into the model of the supervision +/- training database that included variables for baseline performance and time since supervision began. The “supervisor provided feedback” variable in this database did not have a univariable p-value < 0.10. However, this variable did have a univariable p-value < 0.10 in the modeling results of the supervision +/- other strategy components database. Therefore, as an exploratory analysis, we forced this variable into the multivariable model of the supervision +/- training database.
  5. Details on eligibility
     1. Inclusion criteria: 1) professional HCP studies (i.e., no lay HCP predominant studies) with at least one comparison of routine supervision versus a no-intervention control, 2) supervision frequency < 12 visits per year (studies with missing frequency were included), and 3) at least one practice outcome expressed as a percentage.
     2. Exclusion criterion: equivalency studies.
  6. Additional details on modeling for the three supervision databases
     - 1. Database of studies on supervision only. Random-effects modeling could not be performed on this database because it contained only nine studies. Therefore, we conducted a modeling analysis using Generalized Estimating Equations. Specifically, we used the SAS GENMOD procedure with the “normal” response probability distribution and “identity” link function to account for clustering of effect sizes within studies. No supervision attributes had a univariable p-value < 0.10, and thus no additional modeling was done.
       2. Database of studies on supervision +/- training (see Table D1). All models included 1 indicator variable for the presence of training.
       3. Database of studies on supervision +/- other strategy components (see Table D2). All models included six indicator variables for the presence of non-routine supervision components: i.e., community supports, strengthen infrastructure, health systems financing and other incentives, other management techniques, training, and any non-routine supervision strategies (e.g., audit with feedback). Six indicator variables for the presence of other non-supervision component categories (i.e., governance or regulation, group problem solving, patient supports, HCP-directed financial incentives, printed information or job aids for HCPs, and information and communication technology for HCPs) were excluded from models because they were highly unbalanced (i.e., one level of the variable had <8 comparisons).
- Direct analysis (head-to head comparisons)
  1. Eligible comparisons were: a) supervision approach A vs. supervision approach B (e.g., monthly versus quarterly supervision), and b) supervision approach A + other strategy components vs. supervision approach B + other strategy components.

Section 2. Additional results

*Section 2a. Detailed flowchart of the literature search of the systematic review on which this study is based*

Figure S1. Detailed flowchart of the literature search

Abbreviation: HCP = health care provider.

^a^ Early in the initial review’s search of on-line document inventories and websites, detailed records were not kept of the number of citations that were screened. Thus, the number of exclusions is unknown; the exact number of records screened is unknown, but was more than 23,265 (which reflects the number once detailed records began to be kept); the exact number of full-text articles assessed is unknown, but was more than 1202 (which reflects the number once detailed records began to be kept); and the exact number of included articles is unknown, but was more than 205 (which reflects the number once detailed records began to be kept).

^b^ Early in the initial review’s search of the bibliographies of the 510 previous reviews and other papers, detailed records of the search were not kept. Thus, the number of exclusions and full-text assessments are unknown; and the exact number of included articles is unknown, but was more than 247 (which reflects the number once detailed records began to be kept).

*Section 2b. Sample size information*

Table S1. Sample size information: analysis of percentage and continuous practice outcomes for study objectives 1 and 2 combined

| **Health care provider category** | **True control comparisons** | **Head-to-head comparisons** | **Total** |
| --- | --- | --- | --- |
| **LHW predominant^a^** | 2 effect sizes  2 comparisons  2 studies | 4 effect sizes  3 comparisons  3 studies | 6 effect sizes  5 comparisons  5 studies |
| **Not LHW predominant** | 274 effect sizes  74 comparisons  68 studies | 58 effect sizes  11 comparisons  10 studies | 332 effect sizes  85 comparisons  78 studies |
| **Total** | 276 effect sizes  76 comparisons  70 studies | 62 effect sizes  14 comparisons  13 studies | 338 effect sizes  90 comparisons  81 studies |

Abbreviation: LHW = lay health worker.

^a^ These studies evaluated the effect of a strategy designed to improve LHW practices, even if other types of health workers were providing services in the study setting.

Note: 1 effect size for a continuous process outcome from a study involving LHWs was an equivalency comparison with a gold standard control group (COMP_IDnew 29130000412: intervention group: removal of supervision (a pre-existing strategy) but HCPs continued to receive reminders (also a pre-existing strategy) vs. control group: supervision and reminders (both pre-existing strategies).

Table S2. Sample size information: analysis of percentage practice outcomes for study objective 1

| **Health care provider category** | **True control**  **comparisons** | **Head-to-head**  **comparisons** | **Total** |
| --- | --- | --- | --- |
| **LHW predominant^a^** | 1 effect size  1 comparison  1 study | 1 effect size  1 comparison  1 study | 2 effect sizes  2 comparisons  2 studies |
| **Not LHW predominant** | 51 effect sizes  16 comparisons  16 studies | 50 effect sizes  11 comparisons  10 studies | 101 effect sizes  27 comparisons  26 studies |
| **Total** | 52 effect sizes  17 comparisons  17 studies | 51 effect sizes  12 comparisons  11 studies | 103 effect sizes  29 comparisons  28 studies |

Abbreviation: LHW = lay health worker.

^a^ These studies evaluated the effect of a strategy designed to improve LHW practices, even if other types of health workers were providing services in the study setting.

Table S3. Sample size information: analysis of continuous practice outcomes for study objective 1

| **Health care provider category** | **True control**  **comparisons** | **Head-to-head**  **comparisons** | **Total** |
| --- | --- | --- | --- |
| **LHW predominant^a^** | 1 effect size  1 comparison  1 study | 1 effect size  1 comparison  1 study | 2 effect sizes  2 comparisons  2 studies |
| **Not LHW predominant** | 6 effect sizes  5 comparisons  4 studies | 8 effect sizes  5 comparisons  5 studies | 14 effect sizes  10 comparisons  9 studies |
| **Total** | 7 effect sizes  6 comparisons  5 studies | 9 effect sizes  6 comparisons  6 studies | 16 effect sizes  12 comparisons  11 studies |

Abbreviation: LHW = lay health worker.

^a^ These studies evaluated the effect of a strategy designed to improve LHW practices, even if other types of health workers were providing services in the study setting.

Table S4. Sample size information: analysis of percentage practice outcomes for study objective 2

| **Health care provider category** | **True control**  **comparisons** | **Head-to-head**  **comparisons** | **Total** |
| --- | --- | --- | --- |
| **LHW predominant^a^** | 0 effect sizes | 2 effect sizes  1 comparison  1 study | 2 effect sizes  1 comparison  1 study |
| **Not LHW predominant** | 253 effect sizes  63 comparisons  58 studies | 0 effect sizes | 253 effect sizes  63 comparisons  58 studies |
| **Total** | 253 effect sizes  63 comparisons  58 studies | 2 effect sizes  1 comparison  1 study | 255 effect sizes  64 comparisons  59 studies |

Abbreviation: LHW = lay health worker.

^a^ These studies evaluated the effect of a strategy designed to improve LHW practices, even if other types of health workers were providing services in the study setting.

Table S5. Sample size and risk-of-bias information for the three databases used in the modeling analysis to identify attributes associated with effectiveness of routine supervision: effect size level (study objective 2)

| **Database of studies that tested supervision only: 36 effect sizes from 9 comparisons from 9 studies** | | | |
| --- | --- | --- | --- |
| Risk of bias category | Frequency | Percent |  |
| Very high | 1 | 2.8 |  |
| High | 24 | 66.7 |  |
| Moderate | 5 | 13.9 |  |
| Low | 6 | 16.7 |  |
|  | | | |
| **Database of studies that tested supervision +/- training: 85 effect sizes from 21 comparisons from 21 studies** | | | |
| Risk of bias category | Frequency | Percent |  |
| Very high | 3 | 3.5 |  |
| High | 55 | 64.7 |  |
| Moderate | 20 | 23.5 |  |
| Low | 7 | 8.2 |  |
|  | | | |
| **Database of studies that tested supervision +/- other components: 253 effect sizes from 63 comparisons from 58 studies** | | | |
| Risk of bias category | Frequency | Percent |  |
| Very high | 40 | 15.8 |  |
| High | 90 | 35.6 |  |
| Moderate | 89 | 35.2 |  |
| Low | 34 | 13.4 |  |

Table S6. Sample size and risk-of-bias information for the three databases used in the modeling analysis to identify attributes associated with effectiveness of routine supervision: study level (study objective 2)

| **Database of studies that tested supervision only: 9 studies** | | | |
| --- | --- | --- | --- |
| Risk of bias category | Frequency | Percent |  |
| Very high | 1 | 11.1 |  |
| High | 4 | 44.4 |  |
| Moderate | 1 | 11.1 |  |
| Low | 3 | 33.3 |  |
|  | | | |
| **Database of studies that tested supervision +/- training: 21 studies** | | | |
| Risk of bias category | Frequency | Percent |  |
| Very high | 3 | 14.3 |  |
| High | 11 | 52.4 |  |
| Moderate | 3 | 14.3 |  |
| Low | 4 | 19.0 |  |
|  | | | |
| **Database of studies that tested supervision +/- other components:** **58 studies** | | | |
| Risk of bias category | Frequency | Percent |  |
| Very high | 17 | 29.3 |  |
| High | 19 | 32.7 |  |
| Moderate | 11 | 19.0 |  |
| Low | 11 | 19.0 |  |

*Section 2c. Descriptive results of included studies*

Table S7. General attributes of included studies

| **Study attribute** | **All studies**  **(N=81)** |
| --- | --- |
| Number of study arms |  |
| 1 | 6 (7.4%) |
| 2 | 67 (82.7%) |
| 3 | 8 (9.9%) |
| Total number of study arms across all studies | 164 |
|  |  |
| Total number of comparisons across all studies |  |
| Strategy vs. true (no intervention) control group | 74 (82.2%) |
| Strategy A vs. Strategy B with no placebo components | 14 (15.6%) |
| Strategy vs. placebo control group | 2 (2.2%) |
|  |  |
| Number of effect sizes per study and comparison |  |
| Median number of effect sizes per study (range) | 2 (1–28) |
| Median number of effect sizes per comparison (range) | 2 (1–28) |
|  |  |
| Study designs |  |
| Pre-post study with randomized controls | 36 (44.4%) |
| Pre-post study with non-randomized controls | 23 (28.4%) |
| Post-only study with randomized controls | 10 (12.4%) |
| Interrupted time series with no controls | 6 (7.4%) |
| Interrupted time series with randomized controls | 5 (6.2%) |
| Interrupted time series with non-randomized controls | 1 (1.2%) |
|  |  |
| Economy of country where study was done |  |
| Low income | 42 (51.8%) |
| Lower-middle income | 29 (35.8%) |
| Upper-middle income | 10 (12.4%) |
|  |  |
| Risk of bias |  |
| Low | 17 (21.0%) |
| Moderate | 17 (21.0%) |
| High | 27 (33.3%) |
| Very high | 20 (24.7%) |
|  |  |
| WHO region where study was conducted |  |
| Africa | 44 (54.3%) |
| Southeast Asia | 14 (17.3%) |
| America | 9 (11.1%) |
| Western Pacific | 7 (8.6%) |
| Eastern Mediterranean | 6 (7.4%) |
| Europe | 1 (1.2%) |
|  |  |
| Year of publication (or date of document for unpublished reports), by decade |  |
| 2010 or later (latest year was 2017)^a^ | 31 (38.3%) |
| 2000–2009 | 35 (43.2%) |
| 1990–1999 | 15 (18.5%) |
|  |  |
| Data collection methods (multiple responses allowed per study) |  |
| Record or chart review | 45 (55.6%) |
| Observation of HCP-patient interaction | 26 (32.1%) |
| Interview with patient or patient’s caretaker | 24 (29.6%) |
| Interview with HCP | 16 (19.7%) |
| Simulated client | 9 (11.1%) |
| Observation of facility | 9 (11.1%) |
| Questionnaire for patient or patient’s caretaker | 6 (7.4%) |
| Questionnaire for HCP (any administration method) | 6 (7.4%) |
| Physical exam of patient by study team | 5 (6.2%) |
| Case scenario | 3 (3.7%) |
| Observation of HCP practices not involving real patients | 2 (2.5%) |
| Interview with administrator | 1 (1.2%) |
| Questionnaire for an administrator | 1 (1.2%) |
| Questionnaire for HCP’s supervisor | 1 (1.2%) |
|  |  |
| Urban vs. rural study setting |  |
| Urban +/- peri-urban areas | 21 (25.9%) |
| Mix of urban and rural areas | 27 (33.3%) |
| Rural areas only | 22 (27.2%) |
| Town +/- rural areas | 4 (4.9%) |
| Unclear or not stated | 7 (8.6%) |
|  |  |
| Data available on strategy cost or other economic evaluation (from either the study reports or responses from investigators) | 28 (34.6%) |

Abbreviations: HCP = Health care provider, WHO = World Health Organization.

^a^ Many reports from 2016 and all from 2017 either were originally identified as unpublished, but were published by the time of the analysis, or were reports that authors or experts provided after the formal literature search had ended.

Table S8. Settings of included studies: places where services were delivered, who owned or operated the service delivery points, and types of health care providers

| **Study attribute** | **All studies (N=81)** |
| --- | --- |
| Places where services were delivered (multiple responses allowed) | |
| Outpatient health facility | 52 (64.2%) |
| Hospital outpatient department | 24 (29.6%) |
| Hospital inpatient wards | 15 (18.5%) |
| Household or community setting | 9 (11.1%) |
| Drug shop | 8 (9.9%) |
| Pharmacy | 7 (8.6%) |
| Non-hospital health facility inpatient ward | 5 (6.2%) |
| School | 1 (1.2%) |
|  |  |
| Who owns or operates the place where services were delivered (multiple responses allowed per study) |  |
| Public or government | 54 (66.7%) |
| Private, for profit | 17 (21.0%) |
| Community | 10 (12.4%) |
| Private, not for profit | 6 (7.4%) |
| Private, profit status unknown or not reported | 5 (6.2%) |
| Public-private partnership | 1 (1.2%) |
| Unclear or not reported | 6 (7.4%) |
|  |  |
| Type of health care providers (multiple responses allowed per study) |  |
| Nurse | 37 (45.7%) |
| Physician | 33 (40.7%) |
| Midwife | 21 (25.9%) |
| Nurse aide | 21 (25.9%) |
| Pharmacist assistant or non-pharmacist drug vendor | 13 (16.1%) |
| Lay health worker | 10 (12.4%) |
| Clinical officer | 9 (11.1%) |
| Paramedic or unspecified non-physician | 8 (9.9%) |
| Pharmacist | 8 (9.9%) |
| Health educator or information officer | 3 (3.7%) |
| Laboratorian | 3 (3.7%) |
| Student | 2 (2.5%) |
| Midwife aide | 1 (1.2%) |
| Health care provider, type unspecified | 5 (6.2%) |
|  |  |
| Lay health worker was the predominant type of health care provider | 5 (6.2%) |

Table S9. Health conditions addressed by included studies

| **Health condition**  **(multiple responses allowed per study)** | **No. of studies with at least one effect size related to**  **the health condition,**  **among all 81 studies** |
| --- | --- |
| Multiple (or all) health conditions | 25 (30.9%) |
| Malaria | 18 (22.2%) |
| Acute respiratory infections | 15 (18.5%) |
| Reproductive health (not pregnancy related) | 13 (16.0%) |
| Pregnancy | 12 (14.8%) |
| Diarrhea | 10 (12.3%) |
| HIV/AIDS +\- other sexually transmitted diseases | 9 (11.1%) |
| Vaccine-preventable illnesses | 6 (7.4%) |
| Newborn health conditions | 5 (6.2%) |
| Sexually transmitted diseases (HIV/AIDS not specifically included) | 5 (6.2%) |
| Child health (not covered by other categories, such was well-baby checks) | 4 (4.9%) |
| Malnutrition | 4 (4.9%) |
| Non-communicable diseases (not covered by other categories, such as asthma) | 4 (4.9%) |
| Tuberculosis | 3 (3.7%) |
| Infection prevention | 2 (2.5%) |
| Other infectious diseases (not covered by other categories, such as appendicitis) | 2 (2.5%) |
| General medicine use | 1 (1.2%) |

Table S10. Practice outcome categories of all 338 effect sizes from the included studies

| **Outcome** | **HCP practice outcome scale** | | **Totals for percentage and continuous outcomes combined** |
| --- | --- | --- | --- |
|  | **Percentage** | **Continuous** |  |
| Assessment | 17 studies  19 comparisons  42 effect sizes | 0 studies  0 comparisons  0 effect size | 17 studies  19 comparisons  42 effect sizes |
| Case management^a^ | 22 studies  23 comparisons  42 effect sizes | 0 studies  0 comparisons  0 effect sizes | 22 studies  23 comparisons  42 effect sizes |
| Chemoprophylaxis | 1 study  1 comparison  1 effect size | 0 studies  0 comparisons  0 effect sizes | 1 study  1 comparison  1 effect size |
| Consultation time | 0 studies  0 comparisons  0 effect sizes | 2 studies  2 comparisons  2 effect sizes | 2 studies  2 comparisons  2 effect sizes |
| Counseling and communication | 21 studies  22 comparisons  102 effect sizes | 1 study  1 comparison  2 effect sizes | 21 studies  22 comparisons  104 effect sizes |
| Diagnosis | 6 studies  7 comparisons  7 effect sizes | 1 study  1 comparison  1 effect sizes | 7 studies  8 comparisons  8 effect sizes |
| Documentation by health care provider | 3 studies  4 comparisons  8 effect sizes | 0 studies  0 comparisons  0 effect sizes | 3 studies  4 comparisons  8 effect sizes |
| Patient dignity | 2 studies  2 comparisons  6 effect sizes | 0 studies  0 comparisons  0 effect sizes | 2 studies  2 comparisons  6 effect sizes |
| Patient visit by health care provider | 0 studies  0 comparisons  0 effect sizes | 2 studies  2 comparisons  2 effect sizes | 2 studies  2 comparisons  2 effect sizes |
| Referral | 3 studies  4 comparisons  5 effect sizes | 0 studies  0 comparisons  0 effect sizes | 3 studies  4 comparisons  5 effect sizes |
| Reporting time by health care provider | 1 study  1 comparison  1 effect size | 0 studies  0 comparisons  0 effect sizes | 1 study  1 comparison  1 effect size |
| Treatment | 39 studies  44 comparisons  100 effect sizes | 6 studies  7 comparisons  9 effect sizes | 41 studies  47 comparisons  109 effect sizes |
| Universal precautions  by health care provider | 2 studies  2 comparisons  6 effect sizes | 0 studies  0 comparisons  0 effect sizes | 2 studies  2 comparisons  6 effect sizes |
| Vaccination | 2 studies  2 comparisons  2 effect sizes | 0 studies  0 comparisons  0 effect sizes | 2 studies  2 comparisons  2 effect sizes |
| Total | 76 studies  84 comparisons  322 effect sizes | 11 studies  12 comparisons  16 effect sizes | 81 studies  90 comparisons  338 effect sizes |

^a^ Outcomes that include multiple steps of the case-management pathway (e.g., correct diagnosis and treatment).

*Section 2d. Assessment of publication bias*

First, we performed a visual inspection of a funnel plot of the 16 study comparisons of a supervision strategy versus a no-intervention comparison group from studies of professional health care providers (Figure B). The effect size for a single study comparison was the median of effect sizes of all practice outcomes expressed as a percentage. Our interpretation was that there was no asymmetry, which suggests that there was no publication bias. Second, we used the statistical test proposed by Egger to identify asymmetry (Egger *et al*. BMJ 1997; 315: 629–34). We fit the following model using ordinary least squares linear regression: the dependent variable was the standard normal deviate (i.e., the effect size divided by standard error) and the independent variable was the precision (i.e., 1/standard error). Evidence of possible publication bias was defined as a p-value < 0.1 of the model’s intercept. We found no evidence of asymmetry (intercept p-value = 0.92).

Figure S2. Funnel plot of 16 study comparisons of a supervision strategy versus a no-intervention comparison group from studies of professional health care providers (results of percentage outcomes)

*Section 2e. Effectiveness of supervision strategies for lay health care providers*

Table S11. Effectiveness of supervision strategies on the practices of lay health care providers

| **Strategies tested^a^** | | | **Outcome scale** | **No. of study comparisons (risk of bias: low, moderate, high, very high)** | **Median MES^b^**  **(range)** |
| --- | --- | --- | --- | --- | --- |
| **Intervention arm** | | **Reference arm** |  |  |  |
| *Routine supervision (equivalency study)* | | | | | |
|  | Telephone reminders to HCPs | Routine supervision plus telephone reminders to HCPs | Continuous | 1 (0, 1, 0, 0) | –151.3 (NA)^c^ |
| *Routine supervision (non-equivalency studies)* | | | | | |
|  | Routine supervision | Controls | Percentage | 1 (0, 0, 1, 0) | 22.6 (NA) |
|  | Routine supervision plus other strategy components | Other strategy components | Percentage | 1 (0, 0, 1, 0) | –1.2 (NA) |
|  | Routine supervision plus other strategy components | Other strategy components | Continuous | 1 (0, 0, 1, 0) | 0.3 (NA) |

Abbreviations: HCPs = health care providers, MES = median effect size, NA = not applicable.

^a^ See Boxes 1 and 2 in the main article for descriptions of the strategies and the comparisons, respectively.

^b^ Effect sizes calculated as the intervention arm improvement minus reference arm improvement.

^c^ Result based on one effect size: outcome = mean number of days that HCPs were overdue for a follow-up visit for a given patient per HCP (i.e., a smaller number of days indicates improved HCP performance). In the intervention arm, the outcome increased from 9.1 to 26.9 days (a relative increase of 195.6%, which indicates worsening performance); and among gold standard controls, the outcome increased from 7.9 to 11.4 days (a relative increase of 44.3%, which indicates worsening performance). Thus, the effect size = 44.3% – 195.6%, which means that performance worsened in both arms, but worsened more in the intervention arm than in the control arm.

***Note:*** Two randomized, controlled trials identified by the review by Gangwani *et al*. (Gangwani MK, Khan RS, Das JK. *Systematic Reviews to inform guidelines on health policy and system support to optimise community health worker programmes: In the context of community health worker programmes, what strategies of supportive supervision should be adopted over what other strategies? Final report to the World Health Organisation Guideline Development Group.* November 2017. Centre for Evidence and Implementation: 2017), but excluded from our analyses because they were published after our data source had ended its literature search, had the following findings:

- **Trial 1:** Singh D, Negin J, Orach CG, Cumming R. Supportive supervision for volunteers to deliver reproductive health education: a cluster randomized trial. Reprod Health. 2016; 13(1):126. https://doi.org/10.1186/s12978-016-0244-7.
  - Intervention arm: “routine supervision + other strategy components” (supportive supervision + ongoing monthly training for 10 months)
  - Reference arm: “other strategy components” (ongoing monthly training for 10 months)
  - HCP practice outcome: % of women who were visited by a Community Health Volunteer (***percentage outcome***)
  - Effect size: 81.0% – 40.0% – (63.0% – 42.0%) = 20.0 %-points
- **Trial 2:** Kaphle S, Matheke-Fischer M, Lesh N. Effect of Performance Feedback on Community Health Workers Motivation and Performance in Madhya Pradesh, India: A Randomized Controlled Trial. JMIR Public Health and Surveillance. 2016;2(2):e169.
  - Study comparison 1:
    - Intervention arm: “routine supervision + benchmarking + other strategy component” (supervision with feedback about counseling + benchmarking + counseling job aid)
    - Reference arm: “other strategy component” (“placebo” supervision with feedback about either case activity or form submission + counseling job aid)
    - HCP practice outcome: Mean duration of counseling per form submitted per week, in minutes (***continuous outcome***)
    - Effect size: 100*((8.3 – 2.0)/2.0 – (4.0 – 2.25)/2.25) = 237.2 %-points
  - Study comparison 2:
    - Intervention arm: “routine supervision + benchmarking + other strategy component” (supervision with feedback about case activity + benchmarking + counseling job aid)
    - Reference arm: “other strategy component” (“placebo” supervision with feedback about either counseling or form submission + counseling job aid)
    - HCP practice outcome: Mean % of registered cases who were visited by Community Health Worker (CHW) per week (***percentage outcome***)
    - Effect size: 39.5 – 17.0 – (37.0 – 22.5) = 8.0 %-points
  - Study comparison 3:
    - Intervention arm: “routine supervision + benchmarking + other strategy component” (supervision with feedback about form submission + benchmarking + counseling job aid)
    - Reference arm: “other strategy component” (“placebo” supervision with feedback about either counseling or case activity + counseling job aid)
    - HCP practice outcome: Mean % of children visited by CHW for whom CHW submitted a form per week (***percentage outcome)***
    - Effect size: 69.0 – 45.5 – (81.0 – 50.0) = –7.5 %-points

*Section 2f. Attributes of routine supervision associated with supervision effectiveness for professional health care providers*

Note 1. Modeling of the “supervision alone” database (using fixed-effects-only modeling with Generalized Estimating Equations [GEE]) without variables for baseline performance and time since supervision began forced into the model revealed that no supervision attribute met the inclusion criterion (i.e., a univariable p-value <0.1). No random-effects modeling was performed.

Note 2. GEE and random-effects modeling of the “supervision +/- training” database without variables for baseline performance and time since supervision began forced into the model revealed that no supervision attribute met the inclusion criterion.

Note 3. GEE modeling of the “supervision +/- training” database with variables for baseline performance and time since supervision began forced into the model revealed that no supervision attribute met the inclusion criterion.

Table S12. Routine supervision attributes associated with supervision effectiveness: random-effects modeling results from studies of supervision with or without training. Baseline performance, time since supervision began, and supervisor gives feedback to health care provider forced into the model.^a^

| **Supervision attribute or other predictor of effectiveness** | **β** | **p-value** | **90% CI for β** | |  |
| --- | --- | --- | --- | --- | --- |
| Intercept | 16.8 | 0.02 | 5.7 | 27.8 |  |
| Training included in strategy | –6.9 | 0.31 | -18.0 | 4.1 |  |
| Supervisors gave feedback to health care providers | 7.9 | 0.22 | -2.6 | 18.4 |  |
| Baseline performance | –0.095 | 0.04 | -0.17 | -0.02 |  |
| Time since supervision began, in months | 0.48 | 0.26 | -0.21 | 1.16 |  |
| Adjusted R^2^ | 0.111 | | | | |
| No. of observations missing | 0/85 (0%) | | | | |

CI = confidence interval

^a^ This analysis included 85 effect sizes from 21 study comparisons.

Table S13. Routine supervision attributes associated with supervision effectiveness: modeling results from studies of supervision with or without other strategy components

| **Supervision attribute or other predictor of effectiveness** | **Model 1: no predictors forced into the model** | | | | **Model 2: baseline performance and time since supervision began forced into the model** | | | |
| --- | --- | --- | --- | --- | --- | --- | --- | --- |
|  | **β** | **p-value** | **90% CI for β** | | **β** | **p-value** | **90% CI for β** | |
| Intercept | 8.2 | 0.08 | 0.6 | 15.8 | 20.0 | 0.01 | 8.4 | 31.5 |
| Community support included in strategy | 0.6 | 0.90 | -6.9 | 8.1 | 3.9 | 0.28 | -1.9 | 9.7 |
| Strengthening infrastructure included in strategy | 2.1 | 0.62 | -4.8 | 9.0 | 3.4 | 0.35 | -2.7 | 9.5 |
| Health system financing or other incentives included in strategy | –5.3 | 0.32 | -13.9 | 3.4 | –3.2 | 0.51 | -11.3 | 4.9 |
| Other management techniques included in strategy | 7.7 | 0.11 | -0.2 | 15.6 | 6.8 | 0.17 | -1.3 | 14.9 |
| Training included in strategy | 4.6 | 0.41 | -4.6 | 13.7 | –2.0 | 0.71 | -10.8 | 6.9 |
| Strategy included a “supervision” component that was not routine supervision (e.g., audit with feedback) | –3.3 | 0.62 | -14.2 | 7.6 | –8.1 | 0.19 | -18.2 | 2.0 |
| Supervisors gave feedback to health care providers | 4.1 | 0.27 | -1.9 | 10.1 | 5.6 | 0.14 | -0.6 | 11.9 |
| Supervisors received supervision | 11.5 | 0.051 | 1.9 | 21.2 | 8.8 | 0.097 | 0.1 | 17.4 |
| Supervisor participated in problem-solving with HCPs | 20.8 | 0.032 | 4.9 | 36.7 | 14.2 | 0.098 | 0.2 | 28.3 |
| Baseline performance | NA | | | | –0.23 | 0.005 | -0.37 | -0.10 |
| Time since supervision began, in months | NA | | | | 0.18 | 0.52 | -0.28 | 0.63 |
| Adjusted R^2^ | 0.140 | | | | 0.266 | | | |
| No. of observations missing | 0/253 (0%) | | | | 22/253 (8.7%) | | | |

Abbreviations:

CI = confidence interval

NA = not applicable, which indicates that a predictor was not included in the model.

Box S1. Study limitations

| - Included studies had heterogeneous methods and contexts, which made them difficult to combine. - Many studies had a high risk of bias. - Many studies had a short follow-up period. - Not all potentially relevant supervision attributes^1^ were abstracted. For example, we did not have data on:   - Strength of the supervisor-supervisee relationship (in terms of trust; open, two-way communication; and fostering team spirit), which has been described as an important determinant of supervision’s effect on HCP performance^2^   - Whether lay HCPs were supervised at health facilities versus their regular work site^1^   - Whether Health Management Information Systems data were used to target supervision to lowest-performing health facilities, or   - Whether supervision was integrated across disease programs, which could reduce costs - One key attribute (supervision frequency or dose) had many missing values. - Misclassification of the presence of supervision attributes might have occurred, as the HCPPR assumed that an attribute was absent if the study report did not explicitly state its presence, unless a study author responded to an inquiry otherwise. - The modeling performed on the “supervision with or without other strategy components” database used a simplistic approach to adjust for the effect of non-supervision components, which was unlikely to remove all confounding. Furthermore, the lack of association for certain supervision attributes in the regression modeling might be explained by unmeasured factors that can influence supervision effectiveness (e.g., a lack of medicines, equipment, and staff; supervisor’s high workload; and insufficient incentives and career development opportunities for supervisors^2^; vast geographic distances to cover; high HCP turnover; and HCP absenteeism that caused HCPs to miss supervision visits^1^). - Findings had narrow generalizability, as most supervision strategies were tested by only a few studies, and these studies were often small in scope, often focused on a narrow set of health conditions, and might not have been integrated into health systems. - Modeling did not adjust for multiple comparisons, so the results reflect hypothesis screening rather than true hypothesis testing. - Cost results should be interpreted with caution because they were based on data from very few studies, we had insufficient detail to properly account for inflation and real exchange rates, and the generalizability of cost data from research projects to implications for programs across diverse settings is unclear. |
| --- |

References:

1. Human Resources for Health 2030. Enhanced Supervision Approaches: Phase 1 Landscape Analysis Findings Report. Technical Report, May 2019. https://hrh2030program.org/enhanced-supervision-landscape-analysis/. Accessed 1 Jun 2020.

2. Bailey C, Blake C, Schriver M, Cubaka VK, Thomas T, Martin Hilber A. A systematic review of supportive supervision as a strategy to improve primary healthcare services in Sub-Saharan Africa. Int J Gynaecol Obstet. 2016; 132(1): 117-25. doi: 10.1016/j.ijgo.2015.10.004.
